# Calcium Induces Long-Term Legacy Effects in a Subalpine Ecosystem

**DOI:** 10.1371/journal.pone.0051818

**Published:** 2012-12-20

**Authors:** Urs Schaffner, Christine Alewell, René Eschen, Diethart Matthies, Thomas Spiegelberger, Otto Hegg

**Affiliations:** 1 CABI, Delémont, Switzerland; 2 Department of Environmental Sciences, University of Basel, Basel, Switzerland; 3 Department of Biology, University of Marburg, Marburg, Germany; 4 Ecosystèmes montagnards, Institut national de recherche en sciences et technologies pour l’environnement et l’agriculture, Saint-Martin-d’Hères, France; 5 Department of Biology, University of Bern, Bern, Switzerland; Jyväskylä University, Finland

## Abstract

Human activities have transformed a significant proportion of the world’s land surface, with profound effects on ecosystem processes. Soil applications of macronutrients such as nitrate, phosphorus, potassium or calcium are routinely used in the management of croplands, grasslands and forests to improve plant health or increase productivity. However, while the effects of continuous fertilization and liming on terrestrial ecosystems are well documented, remarkably little is known about the legacy effect of historical fertilization and liming events in terrestrial ecosystems and of the mechanisms involved. Here, we show that more than 70 years after the last application of lime on a subalpine grassland, all major soil and plant calcium pools were still significantly larger in limed than in unlimed plots, and that the resulting shift in the soil calcium/aluminium ratio continues to affect ecosystem services such as primary production. The difference in the calcium content of the vegetation and the topmost 10 cm of the soil in limed vs. unlimed plots amounts to approximately 19.5 g m^−2^, equivalent to 16.3% of the amount that was added to the plots some 70 years ago. In contrast, plots that were treated with nitrogen-phosphorus-potassium fertilizer in the 1930s did not differ from unfertilized plots in any of the soil and vegetation characteristics measured. Our findings suggest that the long-term legacy effect of historical liming is due to long-term storage of added calcium in stable soil pools, rather than a general increase in nutrient availability. Our results demonstrate that single applications of calcium in its carbonated form can profoundly and persistently alter ecosystem processes and services in mountain ecosystems.

## Introduction

While intensive forms of land-use are essential for humans to obtain natural products such as food and fibre, they have contributed to the transformation of ecosystem patterns and processes [1]. In particular, human activities have altered the rate, pathways and efficiency of the movement of nutrients within and between the various biotic or abiotic ecosystem compartments. These changes in nutrient cycles do not only affect today’s ecosystem functioning but may also result in long-term legacy effects on ecosystem processes, thereby changing the resilience of ecosystems and their adaptive capacity to sustain ecosystem services in the face of uncertainty and global change [2], [3], [4].

So far, most emphasis in terms of terrestrial nutrient cycling has been put on carbon (C), nitrogen (N) and phosphorus (P), because of their key roles in primary production and climate regulation [5], [6]. For example, the effects of continuous fertilization of grasslands [7], [8], [9], [10], and to some extent also the changes after cessation of fertilization [11], [12], [13], have been well documented. Less well understood are the short-term and long-term effects on ecosystem patterns and processes of the addition of elements other than C, N or P, although they are also known to affect ecosystem functioning. Calcium (Ca) is a key base cation in the soil influencing various physiological and structural processes both in animals and plants [14]. Ca is a component of the soil buffering system, as CaCO3– HCO3- is an important sink for protons with a high buffering rate. Furthermore, Ca as a bivalent cation with a rather strong sorption capacity is very effective in binding to soil particles, thereby increasing aggregate formation in soils and improving soil structure significantly, and an important early indicator of disturbance of nutrient cycling in terrestrial ecosystems [14]. It also affects soil pH and consequently all major soil processes that are modulated by soil pH. Large amounts of lime (CaCO_3_) are applied to croplands, pastures and forests in many regions of the world [15], [16], [17], and they are expected to further increase globally due to the expansion of intensive land-use practices [18]. While liming can improve plant health in naturally or anthropogenically acidified soils [19], [20], it may also contribute to the loss of rare and threatened species [11], [16], [21] and is discussed controversially as either decreasing [22] or increasing the CO_2_ sink capacity of soils [23]. Processes that influence Ca availability are therefore highly relevant to ecosystem regulation and management [20], [23], [24].

In the early 1930s, a unique long-term experiment was set up at the Schynige Platte, Bernese Oberland (Switzerland), to improve site productivity and fodder quality and vegetational composition of subalpine grasslands [25]. Natural acidification of the top soil layers through leaching of cations is a common process in subalpine grasslands of the European Alps, since precipitation exceeds evapotranspiration considerably [14]. On the experimental site at the Schynige Platte, liming and NPK (nitrogen-phosphorus-potassium) fertilization were applied for a period of 2–4 years. Initially, both treatments had strong effects on vegetational composition and led to a significant increase in primary production [25]. Revisiting the experimental site more than 70 years after the last applications had been made, Spiegelberger et al. [11] found that limed plots continued to differ significantly from control plots in the composition of the soil microbial community and the vegetation, with a significant reduction in abundance of several rare plant species. In contrast, the initially strong effects of NPK fertilization on vegetation composition and primary production were not detectable any more [11].

Here we set out to compare the chemical composition of below- and above-ground ecosystem compartments of limed, fertilized and control plots. We focused our analyses on cations indicating increased nutrient availability, i.e. calcium (Ca), potassium (K) and magnesium (Mg), and on indicator cations of nutrient-poor acid soil conditions, i.e. aluminium (Al), iron (Fe) and manganese (Mn). We also analysed total concentrations of Ca, N, and C as well as soil organic matter, and we assessed whether primary production is correlated with concentrations of Ca or N, soil pH or the Ca/Al ratio. The latter is important for soil processes, since free ionic Al is an important determinant of acidity stress because of its toxic effects on plant as well as microbial cell physiology, and Ca can compete with Al at cell membrane binding sites [14], [26], [27]. The concentration of Ca in the top 10 cm soil and in the above-ground vegetation was also used to estimate the difference in total Ca content between limed and control plots. We hypothesized that a) a significant amount of the Ca added more than 70 years ago had been retained in the soil-vegetation loop, despite the fact that cation leaching is a well-known process in subalpine grasslands, and that b) liming induced a shift towards either a generally higher nutritional level or a reduced acidity stress in the soil. Based on our results, we propose a possible mechanism for the long-term legacy effects of historical liming in this subalpine ecosystem.

## Results

**Figure 1:**
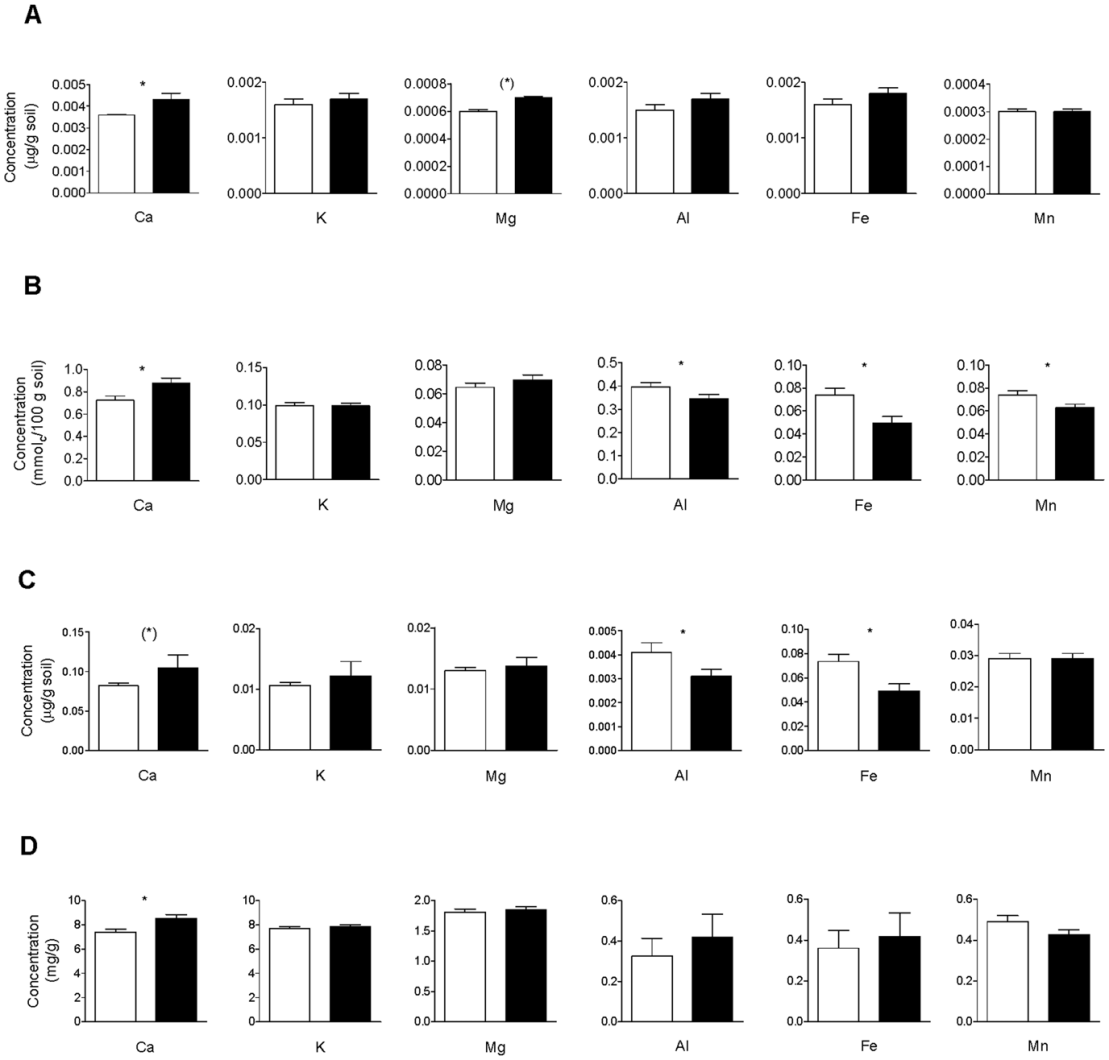
Cation concentrations in soil compartments and in above-ground vegetation of the subalpine grassland at Schynige Platte. A) Soil solution, B) soil cation exchange capacity, C) soil microbial community, and D) above-ground vegetation. Ca = calcium, K = potassium, Mg = magnesium, Al = aluminium, Fe = iron, Mn = manganese. Means +1 SE. Black bars indicate limed plots, white bars unlimed plots (*, P<0.05; (*), P<0.1).

We found that limed and unlimed plots, 70 years after treatments were applied, still differed significantly in their Ca concentrations in the soil soluble fraction (F_1,73_ = 4.028; P = 0.048), the soil cation exchange capacity (F_1,73_ = 6.064; P = 0.016), as well as in total Ca concentration in the soil (F_1,73_ = 7.431; P = 0.008) and in the above-ground plant biomass (F_1,73_ = 7.524; P = 0.008). Ca concentration in the soil microbial fraction was marginally higher in limed plots (F_1,73_ = 3.364; P = 0.071) (Fig. 1; Table 1). Plots that had been treated for two years and plots that had been treated for four years did not differ significantly in Ca concentration in the different soil compartments or in the above-ground plant biomass (all P>0.1). Interestingly, with the exception of a marginally higher Mg concentration in the soil solution (F_1,73_ = 3.302; P = 0.073), the concentration of other nutrient elements that are indicators of improved nutrient availability under neutral or basic soil conditions, such as N, P and K, did not differ between limed and unlimed plots (Fig. 1; Table 1). In contrast, the percentage of exchangeable Al (F_1,73_ = 5.113; P = 0.027;), Fe (F_1,73_ = 4.610; P = 0.035) and Mn (F_1,73_ = 5.757; P = 0.019) were significantly lower, and the concentration of Al (F_1,73_ = 4.427; P = 0.039) and Fe (F_1,73_ = 8.113; P = 0.006) was also lower in the microbial fraction of limed plots. The soil C concentration did not differ between limed and unlimed plots (Table 1).

**Table 1:** Vegetation and soil characteristics in limed and unlimed plots.

Fraction	Limed	Unlimed	F_1,73_	P
Above-ground biomass(g m^−2^)	117.0±0.90	95.8±0.57	4.250	0.043
Soil organic mattercontent (%)	16.0±0.4	17.2±0.9	3.591	0.062
Soil Ca concentration (‰)	2.05±0.08	1.78±0.07	7.344	0.008
Soil C concentration (%)	7.33±0.17	7.72±0.23	2.135	0.148
Soil H concentration (%)	1.43±0.03	1.48±0.03	1.244	0.268
Soil N concentration (%)	0.60±0.01	0.62±0.01	2.404	0.125

Mean values ±1 SE are shown.

In contrast to limed plots, the plots treated with NPK fertilizer did not differ from the control plots in the concentration of Ca, Mg, K, Al, Fe or Mn cations in any of the ecosystem compartments analysed, or in soil total Ca, N or C concentrations (all P>0.2). Also, whether lime was added in the 1930s in combination with NPK fertilizer or alone did not affect cation concentrations in the different soil fractions or in the vegetation (for all interactions NPK×lime P>0.2).

In the early 1930s, each of the plots received either 80 g Ca m^−2^ (two years of application) or 160 g Ca m^−2^ (four years of application), respectively [26]. The results from total soil digestion and from vegetation analysis indicate that limed plots contained 143.5 g Ca m^−2^ in the top 10 cm of the soil and ca. 3.0 g Ca m^−2^ in the vegetation, while unlimed plots contained 124.6 g Ca m^−2^ in the soil and 2.4 g Ca m^−2^ in the vegetation. Hence, to date the average difference in Ca content in limed vs. unlimed plots in the top 10 cm of the soil amounts to 18.9 g m^−2^, and that in the vegetation to 0.6 g m^−2^. The sum (19.4 g Ca m^−2^) is equivalent to 16.3% of the average amount of Ca that had been added to the soil in the early 1930s. Based on the differences in productivity and in Ca concentration of the above-ground biomass between limed and unlimed plots (Fig. 1; Table 1), annual removal of the above-ground biomass led to a 0.28 g higher loss of Ca in limed than in unlimed plots in 2006. Assuming that the differences in productivity and Ca concentration of the above-ground biomass between limed and unlimed plots have been relatively stable over the course of the 71 years between the last lime application and the year of soil and vegetation sampling, annual removal of the above-ground vegetation led to a higher loss of Ca on limed plots of c. 20.2 g Ca m^−2^, equivalent to 16.8% of the Ca that had been added in the early 1930s. It should be noted, though, that these numbers are estimates based on a one-time sampling and should therefore be treated with caution; however, since the productivity of limed plots in the early years after the treatments were significantly higher than 70 years later [25], the numbers may be considered as conservative estimates.

**Figure 2:**
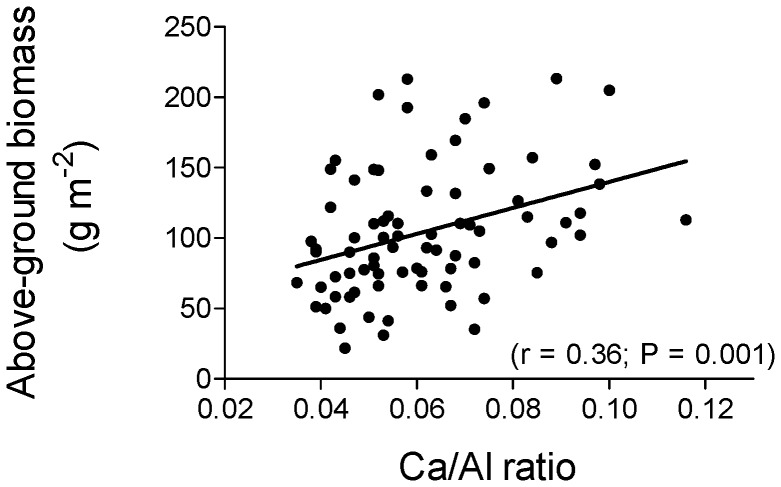
Relationship between the calcium to aluminium (Ca/Al) ratio in the soil and annual above-ground primary production in the subalpine grassland at the Schynige Platte.

Annual primary production increased significantly with the Ca/Al ratio (Fig. 2), but was not related to total soil Ca or N concentration or to soil pH (all P>0.3).

## Discussion

Our results indicate that a significant amount of the added Ca is still present in the top soil layer and in the vegetation more than 70 years after the last liming treatment. Little is known about the migration velocity of Ca in the soil, but our findings are consistent with results from studies investigating the migration of ^90^Strontium, an ion generally believed to behave very similar to Ca in soils and plants. For example, Bossew et al. [28] concluded that the migration velocity of ^90^Strontium within the soil profile is between 0.1 and 2 mm yr^−1^ in a sandy soil. Assuming Ca to have a similar migration pattern, the major fraction of Ca should not have migrated more than 14 cm in depth. Since the soils at Schynige Platte have a higher content of silt, clay and organic matter than those studied by Bossew et al. [28] and thus have a higher sorption capacity and affinity for Ca, migration should be even slower. Of the added Ca still stored in the soil only 15% was found in the exchangeable Ca fraction. The latter is noteworthy, because this implies that the major part of the Ca is, either organically or as a mineral non-exchangeable fraction, bound in the soil. However, observed differences in vegetation between limed and unlimed plots are nevertheless likely to be related to differences in Ca.

There are two possible explanations for the long-term effects of liming: 1) Liming induced a general shift towards a higher nutritional level of the soil resulting in a relatively stable new state for the whole ecosystem (considering that the effects of 2–4 years of liming are still visible after more than 70 years), or 2) the added Ca is stored in stable “permanent” forms and thus not easily leached from the soil. Even though the soil pH is still enhanced in limed plots [11], no effect was observed on the other macro-nutrients (Mg, P, N, K). Thus, our data do not provide evidence that the whole system shifted towards a state of higher nutritional status. Rather, our findings suggest that long-term storage of the added Ca in relatively stable soil pools is responsible for the long-term legacy effect of single liming events on this subalpine ecosystem. As already observed by Lüdi [25], liming favoured plant species that have high rates of nutrient acquisition and high relative growth rates. We propose that a significant amount of lime that was applied in the 1930 was incorporated in plant or microbial biomass. The dead, Ca-enriched biomass that remained in the field was then incorporated into the soil organic matter pool, thereby increasing the Ca fraction that was more permanently bound. Since mountain grasslands can, despite the dominance of perennial species, undergo relatively rapid species turnover at small scales [29], we propose that the current plant species composition [11] is still affected by the altered soil Ca levels in limed plots. This implies that the more permanently bound Ca fractions are available to plants and/or microorganisms, or are affected by longer term weathering. The exchangeable cation pool in soils is a very old concept in soil science dating from the beginning of the 20^th^ century [30]. Several studies have challenged the concept and asked for a redefinition considering (i) organically bound nutrient cations, especially in acid soils [30], and/or (ii) the existence of pools available to plant and microorganisms other than the ‘exchangeable pool’ [15], [31]. It has been suggested, that Ca can be made directly available from rocks, most likely via mycorrhizal fungi, which are able to dissolve Ca from Ca feldspars and apatite [31], [32]. Hence, plants with their symbiotic fungi in the limed plots might be able to take up Ca from non-exchangeable pools in the soil, thereby refilling the exchangeable Ca pool, or they use Ca from the exchangeable pool that is continuously refilled through weathering/decomposition of the Ca enriched ‘non-exchangeable fraction’.

A very profound effect of the liming was an increase in soil pH from 4.5 to almost 7 within a few years after the treatments [25]. The soil pH has decreased considerably since, but is still higher than that of the unlimed plots (pH_soil_ 5.14±0.04 vs. 4.97±0.04) [11]. Soils at the Schynige Platte are deeply leached and strongly acidified overlying limestone. The predominant buffer mechanism in these soils is not the CaCO_3_ system but dissolving Al oxides and hydroxides that release free ionic Al into the solution. The latter is potentially toxic to plants as well as microorganisms, most likely because of inter-ionic competition at cellular membranes. Thus, even if acidified soils have low Ca contents, a general Ca deficiency of plants is unlikely because of the low demand of plants for Ca [33]. However, since Ca can compete with Al at the cell membrane binding sites, the Ca/Al ratio in solution might be a better indicator of the Ca nutritional status of soils or the Al toxicity stress for plants than the Ca concentration [15], [26], [27]. In a recent review, Vanguelova et al. [27] concluded that the fine root Ca/Al ratio has been repeatedly found to be positively correlated with seedling growth and nutrient uptake in woody plant species. The fact that in our experimental site the Ca/Al ratio, but not other measures such as soil pH, explained a significant amount of the variation in annual primary production suggests that a low Ca/Al ratio might also be a suitable indicator of soil acidity stress for plants in acidified mountain grasslands.

While single applications of NPK fertilizer initially showed a strong effect on productivity and vegetation composition [25], there were no effects of historical fertilization on the chemical composition of any of the below-ground and above-ground compartments analysed 70 years after the last treatment. This suggests that the duration of a legacy effect of historical human activities strongly depends on the type of activity or disturbance and that Ca may, at least in acidic subalpine grassland soils and under addition rates comparable to those used in this experiment, cause a more long-term legacy effect than N and P. Despite the fact that P was added as superphosphate, which also includes Ca, the soil pH of fertilized plots only moderately and temporarily increased from 4.5 to 4.7 immediately following application of the treatment [25].

Our study provides unique experimental evidence of the long-term memory of subalpine soils to single events of liming. Despite the small size of the experimental plots and the continuous removal of the above-ground biomass, the Ca-concentration in all ecosystem compartments investigated differed between plots that were limed in the 1930s and unlimed plots, and these patterns continue to affect ecosystem processes. While the increase in primary production by the legacy of the liming treatment may be considered beneficial from an economic point of view, it has detrimental effects on biodiversity since it led to a replacement of rare species by ubiquitous species adapted to increased nutrient availability [11]. Thus, from a general perspective, our findings suggest that small-scale human and natural perturbations might cause long-lasting small-scale patterns in nutrient cycling and hence influence below-ground and above-ground community composition in acid mountain grasslands [11]. From the more specific view of sustainable ecosystem and soil management, the results indicate that the widely used practice of soil liming might have long-lasting effects on plant and soil microbial biodiversity as well as on the chemical composition of soils (pH_soil_, Ca and Al content), at least for several decades or even centuries. Long-lasting legacies of human activities might be particularly common in mountain ecosystems, which are characterized by low rates of nutrient cycling [34]. The management of mountain ecosystems should therefore be based on a thorough understanding of the interrelationships between the legacy effects of historical land-use, ecosystem resilience and the sustainability of socio-ecological systems in order to sustain or restore desirable ecosystem patterns and processes in the face of global change [4].

## Materials and Methods

### Site Description

The experiment at the Schynige Platte near Interlaken (Bernese Oberland, Switzerland) was set up in 1930 by Werner Lüdi at 1925 m a.s.l. in a subalpine acid grassland (acid cambisol, pH at the beginning of the experiment: 4.5–5.0) [25] on a SSE-facing slope with an inclination of 20°. Mean annual precipitation is c. 1800 mm, and mean annual temperature is c. 1°C. Before 1930, the site had been used as a pasture for many centuries. Once the experimental plots had been set up and the treatments initiated, the whole site was fenced and was mown once a year at the end of the growing season in September. From 1958–1980 the site was grazed by cattle. Since 1980, the site has been fenced again and mown once a year. The vegetation of the site consisted mainly of grasses, in particular *Nardus stricta* L. and *Festuca rubra* L. with a mean cover of approximately 30% and 5%, respectively. Subordinate species with a mean cover of between 3% and 5% were *Arnica montana* L., *Crepis conyzifolia* (Gouan) Kerner, *Gentiana purpurea* L., and *Vaccinium myrtillus* L. [11].

In the early 1930s, a total of 340 plots of 2.56 m^2^ each (1.6×1.6 m, separated by 40 cm wide access paths) were set up in blocks and were subjected to different treatments [35]. To assess the effect of liming and NPK fertilization on soil nutrient cycling, we analysed the 80 plots corresponding to experimental blocks 8–11.

Each block consisted of 20 plots which were arranged in two parallel rows of 10 plots along the main altitudinal gradient. The plots were subjected during two years (1932 and 1933) in a factorial design to the two treatments liming (yes/no) and NPK fertilizer (yes/no). Both treatments were applied once a year in early summer: N was applied as ammonium sulphate, P as superphosphate and K as 30% potassium chloride [35]. The fertilized plots also received a small amount of Ca as part of the P-fertilizer. In half of the blocks, the treatments were repeated in 1934 and 1935. In total, plots that were limed over a period of two years received 80 g m^−2^ of Ca, and plots that were fertilized received 1.4 g m^−2^ of N, 4.9 g m^−2^ of P, 9.7 g m^−2^ of K, and 18.7 g m^−2^ of Ca. Plots that were treated over a period of four years received twice the amount of nutrients. Within blocks, the four treatment combinations were allocated to the plots in an alternating order.

### Soil and Vegetation Sampling

Soil samples were collected in June 2006 from each plot by taking one soil core (1.8 cm diameter and 10 cm deep) from each corner of the plot. The four subsamples from each plot were pooled for analysis, resulting in approximately 60 g of soil for each plot. Samples were kept for six months at 2°C until analysis. Prior to analysis, all samples were passed through a 2 mm sieve and the plant parts removed.

Above-ground biomass was sampled in early September from the central 1 m^2^ of each plot using a bar mower of 1 m width. The biomass samples were dried at 60°C for 36 h and weighed. The exact surface of the cut area was determined by measuring the length of the area cut by the bar mower. The dried bulk samples were ground using a grass mill and two subsamples of c. 1 g each were taken from each sample. The C, H and N content of the two subsamples was analysed with a CHN analyser at 1050°C (Leco CHN-1000, Leco Corporation, St Joseph, MI, USA), and the results averaged.

Water-soluble cations were extracted by adding 10.0 ml millipore water to 5.0 g of soil and leaving this for 24 h, during which the mixture was manually stirred several times. After 24 h, the mixture was filtered through a 45 µm membrane filter (Whatman ME25, Schleicher and Schuell, Dassel, Germany). The amount of cations in the filtrate was determined at the day of filtration using an ICP Spectrometer (Spectro Ciros Vision, Spectro GmbH, Kleve, Germany). To extract cations from the microbial biomass, the soil was autoclaved (35 min at 126°C) in a steam sterilizer (All American 25X, Wisconsin Aluminium Foundry Co. Inc., Manitowoc, WI, USA). The amount of cations in the autoclaved soil was then determined in the same way as the water-soluble cations. The amount of cations in the microbial biomass was calculated by subtracting the amount of water-soluble cations in untreated soil from the amount of cations in the autoclaved soil. The total amount of cations in the soil was determined by ICP-OES (Ciros Vision; Spectro, Kleve, Germany) after fusion of the powdered samples with Li_2_B_4_O_7_ and dissolution of the melt in 1M HNO_3_. Chemical analysis of the solution was done with ICP-OES. To determine the cation exchange capacity, 2.5 g of soil was soaked overnight in 1 M NH_4_Cl. The day after, 100 mL of 1 M NH_4_Cl was allowed to percolate through the soil for at least 4 h. The resulting soil extract was filtered through a filter paper (LS 14, Schleicher and Schuell, Dassel, Germany) and 1 M NH_4_Cl was added to make the volume of each sample 100 mL [30]. The amount of cations in the extract was analysed in an ICP spectrometer as described above. Water content of the sieved soils was determined gravimetrically by weighing fresh soil and reweighing the soil sample after drying it at 40°C for 36 h. Organic matter content of the dried soil was determined by weighing the soils again after burning at 550°C for 8 h. To determine the total C, H and N content of the soil (% of dry weight), the dried soils were ground and C, H and N content was determined in approximately 200 mg of ground soil in a CHN analyser as described above. Soil pH measures were taken from Spiegelberger et al. [11].

To calculate the Ca content of soil in limed and unlimed plots, we used average soil bulk density reported for subalpine grasslands in the same region of the Swiss Alps and at the same altitude (approximately 0.7 g cm^−3^) [36]. To calculate total Ca content of the vegetation, the belowground vegetation biomass was estimated to be approximately three times as high as the above-ground biomass [36] and the Ca concentration in belowground vegetation biomass to be 20% lower than in above-ground biomass [37], [38].

### Statistical Analysis

Data from the 80 plots were analysed using ANOVA with block as a random factor and lime and fertilizer treatments as fixed factors. We did not apply a Bonferroni correction, because it is considered as overly conservative [39], [40]. Out of 48 analyses 8 tests (17%) were significant at least at the 5% level (compared to 2.4 expected by chance alone), and three of these at the 1% level (compared to 0.48 expected by chance alone). All statistical analyses were done using SPSS version 16.0.
